# Ultrasonic Processing Induced Activity and Structural Changes of Polyphenol Oxidase in Orange (*Citrus sinensis* Osbeck)

**DOI:** 10.3390/molecules24101922

**Published:** 2019-05-18

**Authors:** Lijuan Zhu, Linhu Zhu, Ayesha Murtaza, Yan Liu, Siyu Liu, Junjie Li, Aamir Iqbal, Xiaoyun Xu, Siyi Pan, Wanfeng Hu

**Affiliations:** 1 College of Food Science and Technology, Huazhong Agricultural University, No.1, Shizishan Street, Hongshan District, Wuhan 430070, China; lijuanzhu2019@hotmail.com (L.Z.); orion_zhu@webmail.hzau.edu.cn (L.Z.); ayeshamurtaza@webmail.hzau.edu.cn (A.M.); y20170022@mail.ecust.edu.cn (Y.L.); junjieli2019@hotmail.com (J.L.); aamirraoiqbal@webmail.hzau.edu.cn (A.I.); xiaoyunxu88@gmail.com (X.X.); drpansiyi.hzau.edu@outlook.com (S.P.); 2 Key Laboratory of Environment Correlative Dietology, Huazhong Agricultural University, Ministry of Education, Wuhan 430070, China; 3 Key Laboratory of Structural Biology, School of Chemical Biology & Biotechnology, Peking University Shenzhen Graduate School, Shenzhen 518055, China; siyuliu@pku.edu.cn

**Keywords:** browning reaction, polyphenol oxidase, ultrasonic processing, structural changes, aggregation

## Abstract

Apart from non-enzymatic browning, polyphenol oxidase (PPO) also plays a role in the browning reaction of orange (*Citrus sinensis* Osbeck) juice, and needs to be inactivated during the processing. In this study, the protein with high PPO activity was purified from orange (*Citrus sinensis* Osbeck) and inactivated by ultrasonic processing. Fluorescence spectroscopy, circular dichroism (CD) and Dynamic light scattering (DLS) were used to investigate the ultrasonic effect on PPO activity and structural changes on purified PPO. DLS analysis illustrated that ultrasonic processing leads to initial dissociation and final aggregation of the protein. Fluorescence spectroscopy analysis showed the decrease in fluorescence intensity leading to the exposure of Trp residues to the polar environment, thereby causing the disruption of the tertiary structure after ultrasonic processing. Loss of α-helix conformation leading to the reorganization of secondary structure was triggered after the ultrasonic processing, according to CD analysis. Ultrasonic processing could induce aggregation and modification in the tertiary and secondary structure of a protein containing high PPO activity in orange (*Citrus sinensis* Osbeck), thereby causing inactivation of the enzyme.

## 1. Introduction

The browning of citrus fruits during storage and juice processing often leads to undesirable flavor and nutritional loss in the final products. The reason for this browning is usually attributed to non-enzymatic browning caused by ascorbic acid degradation [1]. The enzymatic browning, which is catalyzed by polyphenol oxidase (PPO), is often ignored with regard to browning reaction in citrus products. This may be due to the presence of a high level of ascorbic acid in citrus fruits, which could reduce the colored quinone to colorless phenol, and therefore prevent the browning reaction from happening [2]. However, in long-term storage, citrus products tend to brown gradually when ascorbic acid is oxidized during storage, and the existing PPO may play its role in and be responsible for the final browning [3,4].

In previous studies, protein with high PPO activity was found in *Satsuma mandarine* juice [3,5]. In this study, PPO with high activity was also found in *Citrus sinensis* Osbeck. These enzymes accumulate in citrus peel, could easily mix with citrus juice during processing and thus catalyze the phenols to quinones, which further polymerize to generate the melanin pigments [6,7]. These colored compounds negatively affect the nutritional and organoleptic qualities, and consequently lower the marketability of citrus products [3].

Conventional methods such as thermal treatments and chemical reagents are mostly used to inhibit the browning [7]. However, thermal processing could even activate the enzyme [3,8], and may also cause loss of quality. Chemical reagents may bring safety problems to the products. Ultrasonic processing is an innovative, non-thermal technology which could retain the quality of food products at mild conditions [9]. Low-frequency, high-intensity ultrasonic processing may effectively enhance the shelf life of the juice product with minimal damage to its quality [10]. The effects of ultrasonic processing on food processing include cavitation bubbles, vibration on shear strength, and temporary generation of spots of extreme physical phenomena, as well as generation of free radicals through sonolysis of water [11]. The ultrasonic energy in liquid causes the formation of cavitation bubbles due to changes in pressure. The collision of these bubbles leads to an increase in temperature (5000 K) and pressure (1000 atm), which may generate turbulence and extreme shear force in the cavitation zone [10].

Numerous studies were conducted to investigate the effect of ultrasonic processing on enzyme inactivation during fruit and vegetable processing [7]. Up to now, literature about PPO inactivation in orange (*Citrus sinensis* Osbeck) juice has rarely been covered. The aim of the current study was to explore the effect of high-intensity ultrasonic processing on the inactivation of PPO and the structural changes of the enzyme through circular dichroism, fluorescence spectral analysis and dynamic light scattering analysis. These structural analyses may explain the mechanism of enzyme inactivation in orange (*Citrus sinensis* Osbeck) juice during ultrasonic processing.

## 2. Results and Discussion

### 2.1. Purity and Molecular Weight

**Figure 1:**
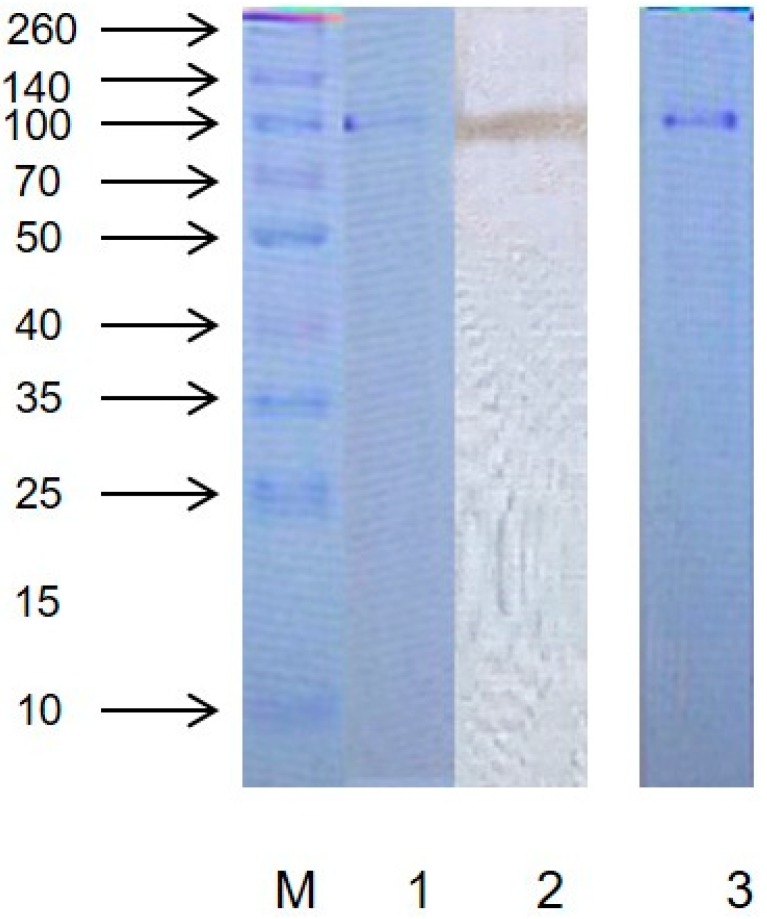
Electrophoresis pattern of the marker (M) and purified enzyme (1—sodium salt (SDS)- Polyacrylamide gel electrophoresis (PAGE) dyed with Coomassie Blue R-250, 2—native PAGE dyed with catechol, 3—native PAGE dyed with Coomassie Blue R-250).

As shown in Figure 1, the purified protein showed only one band after staining with R-250 Coomassie Brilliant Blue by non-denaturing (native) polyacrylamide gel electrophoresis (PAGE), which demonstrated that the protein was electrophoretically pure. Elution profile of protein extraction are shown in Figure S1. The protein purification fold and yield are showed in Table S1. The gel stained with pyrogallol showed the same protein band in the same position, confirming that the protein had PPO activity. As shown in Figure S2, it can also be confirmed that the protein had PPO activity. The protein band coincided approximately with the known protein marker of 100 kDa on the SDS and native PAGE. These results showed that the PPO might be a monomer revealing one band during electrophoresis [12]. Literature demonstrated that PPO from present molecular weight varied from 30 kDa to 128 kDa [13,14,15].

### 2.2. Effect of Ultrasonication on PPO Activity

**Figure 2:**
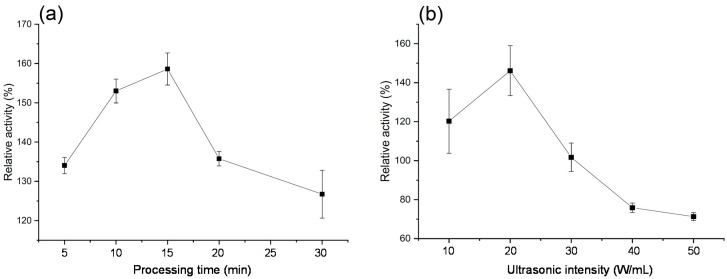
Relative activities of ultrasonic-processed purified PPO at 20 W/mL for 5, 10, 15, 20 and 30 min (**a**); processed for 20 min at 10, 20, 30, 40 and 50 W/mL (**b**).

The PPO activities of the ultrasonicated protein solutions are presented in Figure 2A,B, respectively. As can be seen from Figure 2A, the PPO activity was very high. Ultrasonic processing at lower duration stimulated the activation of PPO. With constant power of 20 W/mL at different times (5, 10, 15, 20 and 30 min) in ultrasonic processing, the relative enzyme activity changed. The PPO activity increased with the increase of time up to 15 min and decreased with a further increase in time. As seen in Figure 2B, with constant ultrasonication for 20 min under different powers, the relative enzyme activity changed. With increasing ultrasonic power, a peak of the relative enzyme activity was observed at 20 W/mL. At above 20 W/mL, the relative enzyme activity began to reduce. Under an ultrasonic power of 40 W/mL, the enzyme activity was restrained; in particular, PPO activity was reduced by 48% under ultrasonic processing at 50 W/mL for 20 min.

These results indicate that low-intensity (20 W/mL or lower) ultrasonic processing stimulates the PPO activity, but high-intensity ultrasonic processing can inhibit the PPO activity. Previous studies demonstrated the accordant effect of ultrasonic processing on PPO activity [7,10,16]. The effect of ultrasonication on the PPO activity may be attributed to the ability of ultrasonic processing to break down the molecular aggregates. Ultrasonic processing at low intensity may dissociate protein monomers which results in the exposure of the active site to the substrate, causing the activation of the PPO enzyme [16]. However, the catalytic center was destroyed under strong ultrasonic processing and more treatment time, which might cause higher levels of denaturation and inactivation of PPO protein.

### 2.3. Particle Size Distribution

**Figure 3:**
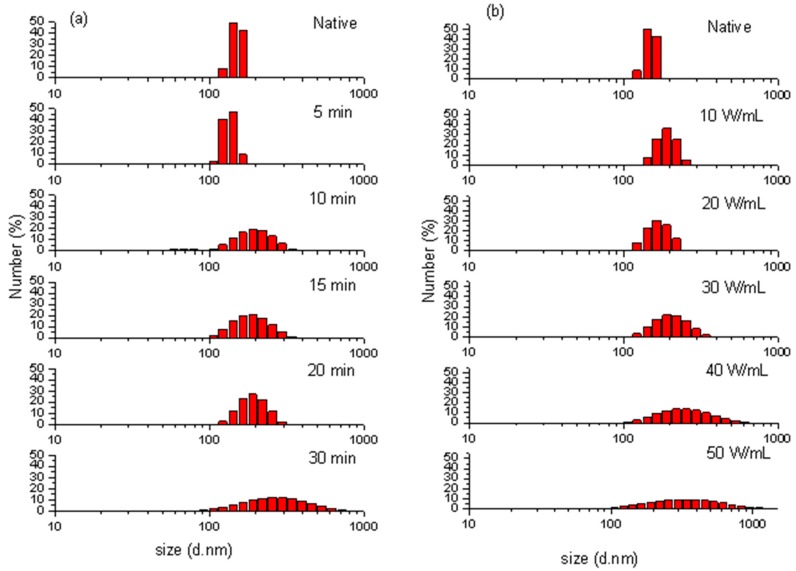
Particle size distributions of native and ultrasonic-processed PPO at 20 W/mL for 5, 10, 15, 20 and 30 min (**a**); processed for 20 min at 10, 20, 30, 40 and 50 W/mL (**b**).

The particle size distributions (PSD) for the untreated and ultrasonicated samples are shown in Figure 3a,b. The untreated protein has a peak value of 49.1% in number fraction at 142 nm with a relatively narrow span of 122 nm to 164 nm (42 nm), which indicates that the native protein aggregate was monodispersed in the aqueous systems [5].

The effect of ultrasonic time (5, 10, 15, 20 and 30 min at 20 W/mL) on the PSD pattern of purified protein is shown in Figure 3a. With ultrasonication for 5 min, the peak particle diameter did not change obviously, but the diameter span increased and the minimum particle diameter decreased. With increased ultrasonic time, the peak particle size and diameter span increased. The maximum peak particle size (255 nm) and diameter span (620.7 nm) were obtained by increasing ultrasonic duration to 30 min. The influence of increasing ultrasonic intensity (10 W/mL to 50 W/mL for 20 min) on PPO is shown in Figure 3b. With the intensity of 10 and 20 W/mL, the peak particle diameter did not obviously change, but the diameter span increased. When the ultrasonic intensity increased to 30 W/mL, the particles started aggregation to attain higher particle diameter. The maximum peak diameter (342 nm) and diameter span (863.7 nm) was obtained after ultrasonic processing at 50 W/mL for 20 min.

In general, after ultrasonic processing, the PSD patterns of protein exhibited remarkable changes with wider spans of larger and smaller particle diameters, suggesting the polydispersion and aggregation of protein particles [17]. The native protein aggregate was monodispersed in the aqueous systems because of hydrogen bonding, hydrophobic interaction and electrostatic interaction. A previous study found that ultrasonic processing caused the cleavage of agglomerates, hydrophobic interaction, and van der Waal’s forces among protein molecules, thereby inducing the structural changes in enzyme protein [18]. In this case, the particle sizes were decreased because of the shear forces due to cavitation [18,19]. However, as the ultrasonic processing duration and intensity increased, some particles started aggregation. Similarly, Gulseren et al. [20] found that ultrasonic processing at a high duration of 40 min could cause the formation of large aggregates in bovine serum albumin protein. This aggregation might be due to the electrostatic and hydrophobic non-covalent interactions among protein particles [20]. The surface hydrophobicity decreased after prolonged sonication on reconstituted whey protein concentrate, which is a sign of protein aggregation [21]. Under the low-intensity or short-term ultrasonic processing, the enzyme exhibited dissociation because of the forces caused by cavitation. However, the aggregation increased with the increased intensity and time due to noncovalent interactions.

### 2.4. Fluorescence Spectroscopic Analysis

**Figure 4:**
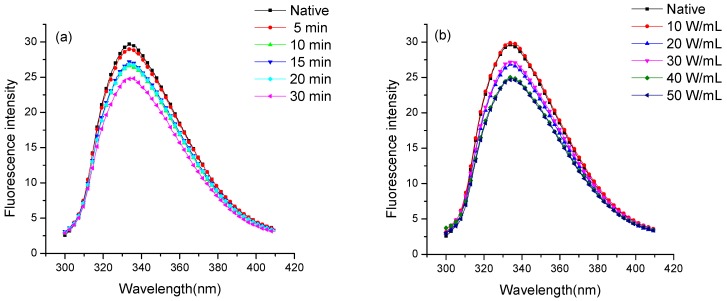
Fluorescence spectra of native and ultrasonic-processed PPO at 20 W/mL for 5, 10, 15, 20 and 30 min (**a**); processed for 20 min at 10, 20, 30, 40 and 50 W/mL (**b**).

The fluorescence property of the native and ultrasonicated purified enzyme was studied by fluorescence spectroscopy. It is a valuable technique to investigate the transition in the tertiary structure of proteins because the fluorescence from the tryptophan of amino acid is sensitive to the polarity of its local environment [16,22]. As shown in Figure 4, the λ_max_ of the native protein was 334 nm with an intensity of 39.74, signifying that Trp residues in enzymes were located in the nonpolar hydrophobic environment. Figure 4a shows the effect of the ultrasonic duration (5, 10, 15, 20 and 30 min at 20 W/mL) on the intrinsic fluorescence spectrum of protein. The ultrasonically treated enzyme showed a red-shift of 0.6–1.2 nm in λ_max_, with a gradual decrease in fluorescence intensity following the increasing treatment duration. This finding indicated the exposure of Trp residues to the polar environment, thereby disrupting the tertiary structure. More exposure of fluorophores to the polar environment might cause the release and transfer of energy, which consequently leads to the quenching of fluorescence intensity [23,24].

Figure 4B illustrates the effect of increasing ultrasonic intensities (10 W/mL to 50 W/mL) on the fluorescence spectra of purified protein. The purified protein showed red-shifts of 0.2–2.0 nm in λ_max_ when treated with low ultrasonic intensities of 10, 20 and 30 W/mL. In the meantime, the fluorescence intensity gradually decreased with increasing ultrasonic intensity. The fluorophores were exposed to a more polar environment due to the polydispersity of protein aggregate after ultrasonic processing as described above, which caused the red-shift and consequently led to the quenching of fluorescence intensity. However, the extreme ultrasonic processing at 50 W/mL induced aggregation and may bury the exposed fluorophores inside the molecules, thereby decreasing the λ_max_. PPO structural change is responsible for the activity change. The changes in fluorescence intensity and λ_max_ indicated a possible change in PPO’s tertiary structure, which ultimately led to the activity reduction of PPO [25]. The result was similar to the observation that the fluorescence intensity of mushroom tyrosinase decreased in an aqueous system following mild thermal and supercritical CO_2_ treatments [24].

### 2.5. Circular Dichroism Spectroscopy Analysis

**Figure 5:**
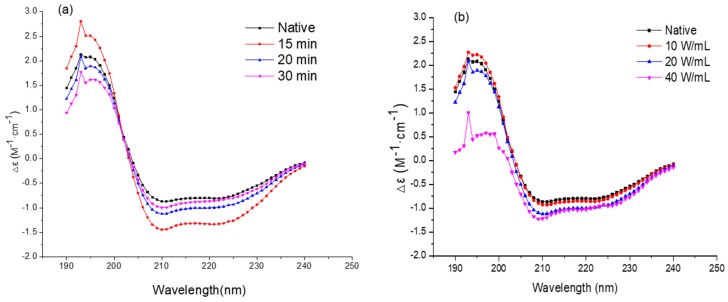
Circular Dichroism (CD) spectra of native and ultrasonic-processed PPO at 20 W/mL for 15, 20 and 30 min (**a**); processed for 20 min at 10, 20 and 40 W/mL (**b**).

The secondary structures of the native and ultrasonicated enzyme were analyzed through CD spectroscopy. The CD spectra of protein are shown in Figure 5a,b. The native protein showed a positive peak at 193 nm with two double-negative slots (208 and 222 nm), which were considered as typical α-helix conformation in the secondary structures [16,26,27]. As shown in Figure 5a,b, the negative peak (208 nm) increased with the negative peak decrease at 222 nm after ultrasonic processing. As the ultrasonic time and intensity increased, the change grew and the two double-negative slots gradually disappeared. These changes show that the ultrasonic processing triggered α-helix conformation of secondary structure loss [3,16,26,27,28]. The contents based on the CONTIN algorithms of the protein secondary structure of the native and ultrasonicated protein [24] are shown in Table 1. At the high ultrasonic intensity of 40 W/mL, the α-helix conformation remarkably decreased, while β-turn contents were increased. A similar study was reported by Liu et al. [10], where ultrasonic processing at high intensity caused a loss of α-helix conformation in protein structure. The decrease in α-helix was found to be correlated with the enzymatic activity of PPO molecules [29]. Ultrasonic processing induced the changes in molecular interaction, thus leading to changes in secondary structure and eventually causing the loss of PPO activity.

## 3. Materials and Methods

### 3.1. Materials and Chemicals

Fresh orange (*Citrus sinensis* Osbeck) used in this study was procured from a local market (Chongqing, China). All chemicals used were of analytical grade.

### 3.2. Extraction and Partial Purification of Protein

Orange tissue (250 g) was homogenized in 700 mL of Tris-HCl buffer (0.5 mol/L) containing 10% polyvinylpyrrolidone (PVPP). The homogenate liquid was stored for 8 h at 4 °C and then subjected to centrifugation at 2057× *g* for 10 min using a centrifuge machine (Eppendorf centrifuge 5804 R, Eppendorf, Hamburg, Germany). The resultant supernatant was fractionated with 25% solid ammonium sulfate to remove impurities. The process was repeated using 90% ammonium sulfate saturation to precipitate proteins with PPO activity. The precipitate was re-dissolved in Tris-HCl (0.5 mol/L) and dialyzed against the same buffer for 34 h. The buffer was changed every hour during the whole process of dialysis. Ultrafilter was used to concentrate the crude extract of protein. Then, DEAE Sepharose Fast Flow and Sepracryl S-200 columns were used to purify the crude protein [5]. The fractions containing the highest activity of PPO were selected, concentrated and stored for further analysis.

### 3.3. Electrophoresis Assay

Native PAGE was carried out on preparative 12% polyacrylamide gels using the method described by Davis [30] with slight modifications. After running, the gels were stained with 0.1 mol/L Catechol (50 mL) and Coomassie Brilliant Blue R-250. The gels were analyzed for activity and estimation of molecular weight. The molecular weight and subunit of purified PPO enzyme were determined by SDS–PAGE [31].

### 3.4. Protein Content

Protein content was determined according to the Bradford method [32]. The protein solution was stained with Coomassie Brilliant Blue G-250 and the absorption peak was observed at 595 nm wavelength [33]. The absorption value of A595 was directly proportional to the protein concentration. The standard curve of bovine serum protein (BSA) was used as a standard protein. The concentration of sample protein was calculated according to the standard curve.

### 3.5. Ultrasonic Processing

An ultrasonic processor (JY92-2D, Ningbo, China) containing a titanium probe of diameter 0.636 cm was used to sonicate 10 mL of protein in 25 mL of centrifugal tubes, surrounded by ice to maintain the low temperature. All ultrasonic processing was definitely below 30 °C. The protein samples were treated at a low frequency of 20 kHz with the pulse duration of 5 s on and 5 s off setting, to investigate the effect of ultrasonic time (10, 15, 20 and 30 min at 20 W/mL) and intensity (10, 20 and 40 W/mL for 30 min) on the protein. The ultrasonic-processed samples were stored at 4 °C for further analysis.

### 3.6. PPO Activity Assay

The activity of the PPO enzyme was measured by determining the increasing rate of absorbance per minute at 420 nm using an Eppendorf Bio-spectrometer (Eppendorf, BioSpectrometer kinetic, Hamburg, Germany). The protein solution of 0.5 mL was mixed with 0.1 mol/L catechol (1 mL) and 0.1 mol/L Tris-HCl buffer (1 mL), then the absorbance of the resultant mixture was measured at 420 nm [5]. The relative activity (RA) of the PPO enzyme was measured according to the following equation.

(1) $$\mathrm{Relative}\;\mathrm{PPO}\;\mathrm{activity}\;=\;\frac{PPO\;activity\;of\;ultrasonic\;treated\;ppo}{PPO\;activity\;of\;native\;ppo}\times 100\%$$

### 3.7. Particle Size Distribution Analysis

Particle size determination was performed using a zetasizer, Nano-ZS device (Malvern Instruments, Malvern, Worcestershire, UK). The purified protein solutions prepared in Tris–HCl buffers (50 mmol/L) were subjected to scattered light wavelength (532 nm) and 15° laser reflection angle at a measuring temperature of 25 °C. The size measurements were taken as the mean of five readings.

### 3.8. CD Spectral Measurement

The purified protein solutions were subjected to CD spectra measurement with a spectropolarimeter (JASCO J-1500, Tokyo, Japan). The samples of treated protein solutions were prepared in Tris–HCl buffer (50 mmol/L) using this buffer as a blank. The secondary structure of protein solutions was determined in the range of far-ultraviolet (196–260 nm) with the scanning speed of 120 nm/min and the bandwidth of 1 nm. Data for CD spectra were presented as changes in the molar extinction coefficient (Δε, M^−1^ cm^−1^). The contents of secondary structure were calculated from the CD spectra by the estimation software of Spectra Manager (JASCO, Japan) [5].

### 3.9. Fluorescence Spectral Measurement

Intrinsic fluorescence spectral measurements were recorded using a fluorescence spectrophotometer F-4600 (Hitachi, Tokyo, Japan). Tris–HCl (50 mmol/L) was used to prepare the protein solution for fluorescence assay. The samples were measured at an emission wavelength of 350 nm to obtain the maximum excitation wavelength and then scanned at this excitation wavelength to record the emission spectra. The *E*_m_ and *E*_x_ slits were set as 5 nm; scan speed was set as 200 nm/min with a response time of 0.1 s [34,35].

## 4. Conclusions

Low-intensity ultrasonic processing stimulated the activation of PPO but exposure to high-intensity ultrasonic processing exhibited an inactivation effect on the PPO enzyme. The increasing power under ultrasonic processing induced the polydispersity of the protein, as well as the change of the secondary and tertiary structures of the protein. The loss of α-helical contents after ultrasonic processing led to the reorganization of the secondary structure of the protein. High-intensity ultrasonic processing caused the exposure of Trp residues to a more polar environment, thereby leading to the quenching of fluorescence intensity and subsequently decreasing the suitability of the catalytic center of the enzyme for reaction with substrates.

## Figures and Tables

**Table 1:** Secondary structure contents of native and ultrasonic-processed PPO.

Samples	α-Helix	β-Sheet	β-Turn	Random Coil
**Native**	76.20%	0.00%	23.60%	0.10%
**10 W/mL/20 min**	76.00%	0.00%	24.00%	0.00%
**20 W/mL/20 min**	53.30%	0.00%	29.30%	17.50%
**40 W/mL/20 min**	32.80%	0.00%	33.60%	33.60%
**20 W/mL/15 min**	54.60%	0.00%	26.50%	18.90%
**20 W/mL/20 min**	53.30%	0.00%	29.30%	17.50%
**20 W/mL/30 min**	51.80%	0.00%	31.10%	17.20%

## Supplementary Materials

The following are available online. Figure S1: Elution profile of protein extraction, Figure S2: Lineweaver–Burk equation of purified enzyme using catechol and pyrogallol as substrates. Table S1: Purification of Polyphenol Oxidase (PPO) from orange (*Citrus sinensis* Osbeck).
