# Supplementary material for: Ultrasonic Processing Induced Activity and Structural Changes of Polyphenol Oxidase in Orange (Citrus sinensis Osbeck)
Source: Molecules. 2019 May 18;24(10):1922. doi: 10.3390/molecules24101922 (PMC6572353; doi:10.3390/molecules24101922)
Supplement: Supplementary file 1 [file molecules-24-01922-s001.zip › Supplementary materials/Figure S2. Lineweaver¿CBurk equation of purified enzyme using catechol and pyrogallol as substrates..docx]

Figure S1. Lineweaver-CBurk equation of purified enzyme using catechol and pyrogallol as substrates.

| 1/[S] | 1/V | |  |  |  |
| --- | --- | --- | --- | --- | --- |
|  | pyrogallol | catechol |  |  |  |
| 25 | 30.81014 | 42.9 |  |  |  |
| 20 | 28.65753 | 38.56 |  |  |  |
| 16.66667 | 26.71741 | 35.74 |  |  |  |
| 14.28571 | 24.41176 | 33.123 |  |  |  |
| 12.5 | 22.56873 | 30.23 |  |  |  |
|  |  |  |  |  |  |
| Protein | Substrate | K_m_ (mol/L) | V_max_ (U/min) | Slope | R^2^ |
|  | Catechol | 0.05218 | 0.05326 | 0.9798 | 0.9861 |
|  | Pyrogallol | 0.04278 | 0.06596 | 0.6485 | 0.9618 |
