# Supplementary material for: Ultrasonic Processing Induced Activity and Structural Changes of Polyphenol Oxidase in Orange (Citrus sinensis Osbeck)
Source: Molecules. 2019 May 18;24(10):1922. doi: 10.3390/molecules24101922 (PMC6572353; doi:10.3390/molecules24101922)
Supplement: Supplementary file 1 [file molecules-24-01922-s001.zip › Supplementary materials/Table S1. Purification of Polyphenol Oxidase (PPO) from orange (Citrus sinensis Osbeck)..docx]

**Table S1.** Purification of Polyphenol Oxidase (PPO) from orange (*Citrus sinensis* Osbeck).

| **Purification Steps** | **Protein Content**  **(mg)** | **Activity**  **(U)** | **Specific Activity**  **(U/mg)** | **Purification Fold** | **Ratio of Recovering Protein (%)** |
| --- | --- | --- | --- | --- | --- |
| **Crude extract** | 600.89 | 305,990.10 | 5.11 | 1 | 100 |
| **90% (NH_4_)_2_SO_4_ Precipitation** | 92.27 | 49,111.41 | 42.66 | 8.35 | 16.05 |
| **DEAE Sepharose Fast Flow** | 5.24 | 3090.50 | 589.79 | 115.42 | 1.01 |
| **Sepracryl S-200 columns** | 1.93 | 1193.24 | 618.26 | 120.99 | 0.39 |
