# Supplementary figures and images for: Ultrasonic Processing Induced Activity and Structural Changes of Polyphenol Oxidase in Orange (Citrus sinensis Osbeck)
Source: Molecules. 2019 May 18;24(10):1922. doi: 10.3390/molecules24101922 (PMC6572353; doi:10.3390/molecules24101922)

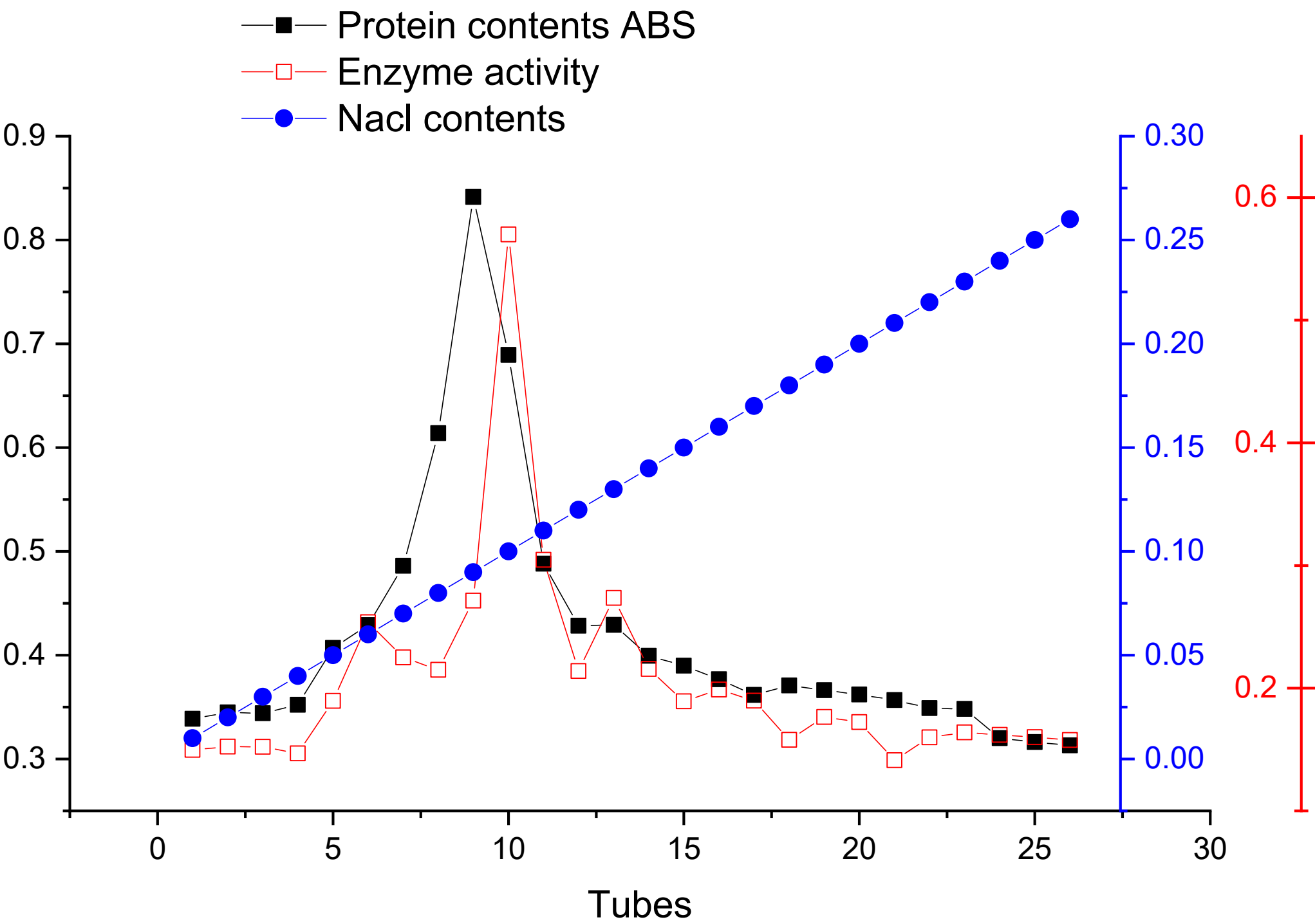

Supplement: Supplementary file 1 [file molecules-24-01922-s001.zip › Supplementary materials/Figure S1. Elution profile of protein extration..pdf]
